# Senolytic Targeting of Bcl-2 Anti-Apoptotic Family Increases Cell Death in Irradiated Sarcoma Cells

**DOI:** 10.3390/cancers13030386

**Published:** 2021-01-21

**Authors:** Julie Lafontaine, Guillaume B. Cardin, Nicolas Malaquin, Jean-Sébastien Boisvert, Francis Rodier, Philip Wong

**Affiliations:** 1 Institut du Cancer de Montréal (ICM), Centre de Recherche du Centre Hospitalier de l’Université de Montréal (CRCHUM), 900 St. Denis Street, Montreal, QC H2X 0A9, Canada; julie.lafontaine.chum@ssss.gouv.qc.ca (J.L.); cardinguill@gmail.com (G.B.C.); nico.malaquin@gmail.com (N.M.); jean-sebastien.boisvert@umontreal.ca (J.-S.B.); rodierf@mac.com (F.R.); 2 Plasma Processing Laboratory, Department of Chemical Engineering, McGill University, 3610 University Street, Montreal, QC H3A 0C5, Canada; 3 Département de Radiologie, Radio-Oncologie et Médicine Nucléaire, Université de Montréal, C.P. 6128, Succursale Centre-Ville, Montreal, QC H3C 3J7, Canada; 4 Département de Radio-Oncologie, Centre Hospitalier de l’Université de Montréal (CHUM), 1051 Sanguinet Street, Montreal, QC H2X 3E4, Canada; 5 Department of Radiation Oncology, Princess Margaret Cancer Centre, 610 University Avenue, Toronto, ON M5G 2M9, Canada; 6 Department of Radiation Oncology, University of Toronto, 149 College Street, Suite 504, Toronto, ON M5T 1P5, Canada

**Keywords:** soft tissue sarcoma, undifferentiated pleomorphic sarcoma, ionizing radiation, pre-operative radiotherapy, senescence, senolytic, ABT-199, ABT-263, BCL-2 family, apoptosis, Venetoclax, Navitoclax

## Abstract

**Simple Summary:**

Limited volumetric change after pre-operative radiotherapy (RT) suggests that sarcomas generally do not undergo cell death. Senolytic drugs represent a highly promising field as a new therapy approach to drive senescent cancer cells towards cell death to enhance treatment response. Here, we demonstrate that the Bcl-2 family of anti-apoptotic proteins in irradiated senescent sarcoma cells represents a senotherapeutic target to improve the cell death response in RT. This study paves the way for new treatment options in soft tissue sarcoma management.

**Abstract:**

Radiotherapy (RT) is a key component of cancer treatment. Most of the time, radiation is given after surgery but for soft-tissue sarcomas (STS), pre-surgical radiation is commonly utilized. However, despite improvements in RT accuracy, the rate of local recurrence remains high and is the major cause of death for patients with STS. A better understanding of cell fates in response to RT could provide new therapeutic options to enhance tumour cell killing by RT and facilitate surgical resection. Here, we showed that irradiated STS cell cultures do not die but instead undergo therapy-induced senescence (TIS), which is characterized by proliferation arrest, senescence-associated β-galactosidase activity, secretion of inflammatory cytokines and persistent DNA damage. STS-TIS was also associated with increased levels of the anti-apoptotic Bcl-2 family of proteins which rendered cells targetable using senolytic Bcl-2 inhibitors. As oppose to radiation alone, the addition of senolytic agents Venetoclax (ABT-199) or Navitoclax (ABT-263) after irradiation induced a rapid apoptotic cell death in STS monolayer cultures and in a more complex three-dimensional culture model. Together, these data suggest a new promising therapeutic approach for sarcoma patients who receive neoadjuvant RT. The addition of senolytic agents to radiation treatments may significantly reduce tumour volume prior to surgery and thereby improve the clinical outcome of patients.

## 1. Introduction

Soft tissue sarcomas (STS) are a group of rare cancers originating from connective tissues, including adipose, fibrous, muscles, neuronal and vascular tissues. These malignancies affect patients of all ages, representing 1% of adult cancers and 7% of pediatric cases [1]. Among patients presenting with primary and non-metastatic STS, the most important prognostic variables are grade, depth, anatomical location and size of the tumour.

A common curative treatment strategy in STS includes surgery and (neo)adjunctive use of radiotherapy (RT). In extremity STS, pre- and post-operative RT have equivalent local control and overall survival outcomes [2,3,4]. Thus, pre-operative RT is often preferred as patients developed fewer long-term irreversible RT complications than those who received post-operative radiotherapy [5,6,7]. Nonetheless, pre-operative RT in STS management seldom results in tumour shrinkage, except for certain subtypes such as myxoid liposarcomas [8,9,10,11], and serves mainly to decontaminate the microscopic diseases surrounding the visible tumour. Clinical evidences of RT’s efficacy in reducing local recurrence (LR) and a lack of rapid tumour shrinkage suggest that RT induces STS cell toxicities without triggering the clearance of damaged cells, as would be expected from apoptotic or necrotic cell death [12]. Modern imaging and RT technologies can deliver accurate and tumour conforming doses, thereby sparing surrounding normal tissues from therapeutic RT doses. Hence, geographical difference in pre-operative RT dose distribution can generate distinct sensitivities and a window of therapeutic opportunity to administer drugs. With an increasing number of molecular agents, some of these may augment the effects of RT [13] and the purpose of this work was to identify new appropriated target agent. Identification of radiosensitizers or methods to accelerate tumour response to RT can reduce tumour volumes, surgical extent and treatment morbidity, and it can potentially render unresectable tumours resectable.

Akin to cytotoxic chemotherapies, RT induces different cellular responses and cell fates, depending on the cancer type or cellular context. Cell fates include autophagy, cell cycle arrest, senescence, mitotic catastrophe, necrosis and apoptosis [14,15,16]. In particular, therapy-induced senescence (TIS) is a well-described response in many cancer types [15,17,18] that is triggered by a variety of stresses, such as RT-induced DNA damage [19]. Senescent cells are characterized by several hallmarks associated with their biological function such as proliferation arrest [20,21], senescence associated beta-galactosidase (SA-β-gal) activity [22,23], persistent DNA damage [24,25] and active senescence associated secretory phenotype (SASP) [26,27,28]. Senescent cells also exhibit an increased resistance to apoptosis [29,30] which, in a cancer treatment context, could result in the ineffective clearance of cancer cells, sustained tumour survival and effects on the tumour microenvironment [31]. Failure to clear senescent cells leads to the chronic accumulation of these cells and has been proposed to support chronic SASP signaling and inflamm-aging [32]. Tissue senescence can also confer a state of stemness in neighboring cells [33,34] and, thus, may create a microenvironment that promotes neoplastic growth, metastasis and immunosuppression [34,35]. Several murine tumour models have also demonstrated the reversibility of TIS, during which epigenetic modelling reprograms TIS cells to acquire enhanced plasticity and stem cell features [33,34]. Correspondingly, TIS has emerged as a potential cell reservoir for treatment resistance and has become a pharmacological target [36,37,38]. Drug inhibition of the Bcl-2 family anti-apoptotic proteins represents one type of senolytic that has demonstrated the ability to specifically induce apoptosis in RT and PARP inhibitor-induced senescent cells and to improve the aging-related functional decline of various organs following senescent cell clearance [37,39,40,41,42].

In this work, we examined the effects of radiation on undifferentiated pleomorphic sarcoma (UPS) cell lines, one of the more frequent and aggressive subtypes of STS, to define their cell fate decisions. This is the first demonstration of RT-induced senescence in STS cell lines and their subsequent sensitivity to Bcl-2 family antiapoptotic protein inhibitors to improve clearance through apoptosis. Our results propose a new therapeutic approach combining RT and senolytic agents to preferentially target senescent STS cells.

## 2. Results

### 2.1. RT Induces Proliferative Arrest in Sarcoma Cell Lines

**Figure 1:**
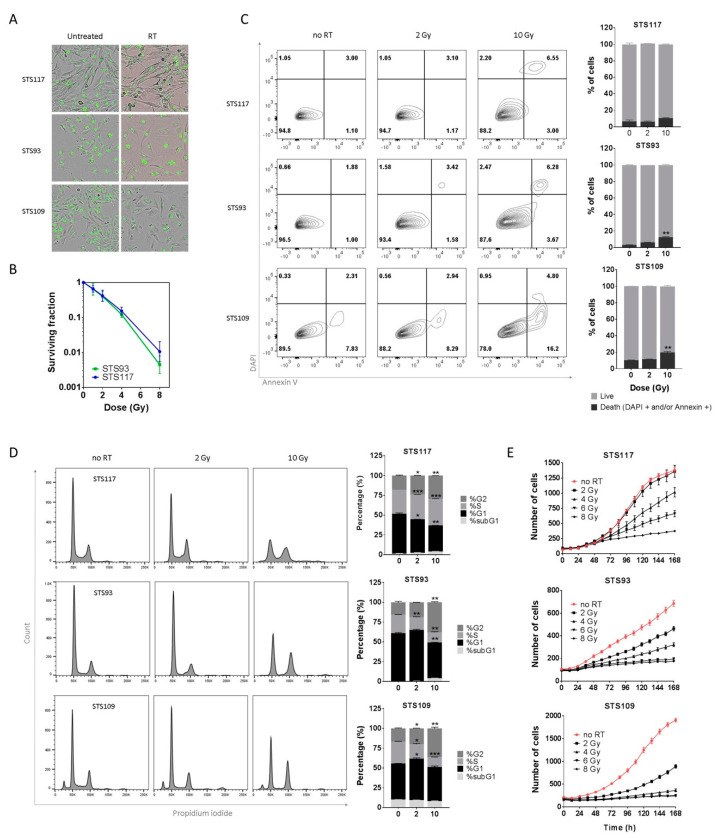
Radiation induces cytostatic effects in sarcoma cell lines. (**A**) Representative picture of the three undifferentiated pleomorphic sarcoma (UPS) cell lines, untreated or 5 days after treatment (8 Gy). (**B**) Clonogenic survival assay of STS93 and STS117 treated with 0, 0.5, 2, 4 and 8 Gy of radiation. Cell survival is normalized to the clonogenic formation from untreated cells (0 Gy). (**C**,**D**) Cell death and cell cycle analyzed by flow cytometry 48 h after exposure to radiation (0, 2 and 10 Gy). In the graph, percentage of cell death (**C**) represents the sum of Annexin V positive cells (both DAPI positives and negatives) and DAPI positive cells (Annexin V negatives) from the quadrant plots of DAPI vs. Annexin V. (**E**) Cell proliferation curves of sarcoma cell lines expressing H2B-GFP and exposed to increasing doses of radiation (0, 2, 4, 6 and 8 Gy). Student’s t-test, * *p* < 0.05, ** *p* < 0.01, *** *p* < 0.001. Data are representative of two to three experiments. Error bars represent ± standard deviation.

To understand the underlying mechanisms involved in the STS cellular response to RT, we characterized the cell fates following RT of three UPS cell lines: STS93, STS109 and STS117. These human cells present distinct morphology, growth properties, genotype and response to drugs or RT treatment [43,44,45] (Figure 1A). Using clonogenic assays, we established the baseline cytotoxic response of STS93 and STS117 cells to RT (Figure 1B). STS109 cells were not included as they did not form colonies. Since clonogenic survival integrates all forms of potential cell death leading to proliferation loss, we then tried to better define their specific therapy-induced cell fates. Cell viability measured by flow cytometry showed only a slight increase in cell death 48 h after RT: less than 10% of STS93, STS109 and STS117 cells underwent apoptosis or necrosis compared to untreated controls basal level (Figure 1C). Correspondingly, cell cycle analyses at increasing RT doses did not allow us to observe a significant variation in the sub-G1 fraction. The cells accumulated in the G2 phase with decreasing cellular fractions in the S phase at higher RT doses (Figure 1D). These observations were consistent with low levels of dying cells and proliferation arrest. Live-cell imaging and monitoring system was used to evaluate cellular responses over a longer period of time (7 days). As expected, a single dose of RT (2, 4, 6 or 8 Gy) was sufficient to decrease the proliferation rate in all cell lines in a dose-dependent manner (Figure 1E). At 8 Gy, cells no longer proliferated (Figure 1E) and changes in cell morphology were observed five days following RT (Figure 1A). Taken together, these results show that RT led to persistent proliferation arrest with minimal STS cell death, which is consistent with the lack of volumetric response to pre-operative RT observed in clinic.

### 2.2. Sarcoma Cell Lines Exhibit a Senescence Phenotype after Treatment

**Figure 2:**
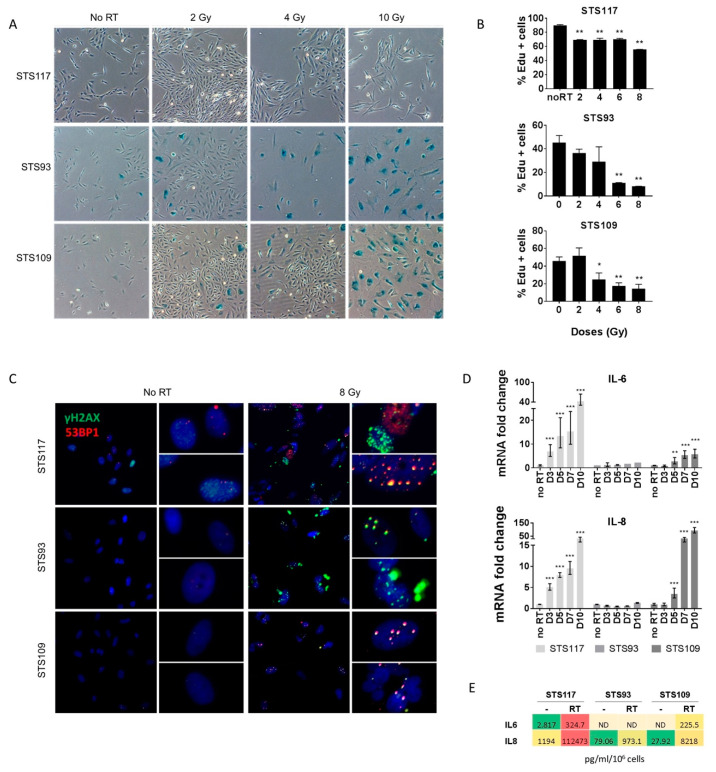
Radiation induces a senescence-like phenotype in sarcoma cell lines. (**A**) SA-β-gal staining 10 days following irradiation (RT) with doses of 2, 4 and 10 Gy. (**B**) Analysis of a 24 h EdU pulse labelling 5 days following radiation (2, 4, 6 or 8 Gy). (**C**) DNA damage associated γH2AX (green) and 53BP1 (red) immunofluorescence 10 days after exposure to 8 Gy. (**D**) mRNA levels of IL-6 and IL-8 over time (D stands for day) relative to untreated controls evaluated by real-time qPCR following 8 Gy of radiation. (**E**) Secretion (pg/mL/10^6^ cells) of IL-6 and IL-8 measured after 10 days (8 Gy). Not Detected (ND) indicated values below the standard curve. Data in (**B**) and (**D**) were analyzed using a two-tail Student’s t-test to compare RT treatment with the untreated control. * *p* < 0.05, ** *p* < 0.01, *** *p* < 0.001. Data are representative of two to three experiments. Error bars indicate ± the standard deviation.

Based on our observations of stable proliferation arrest, we hypothesized that STS cell lines underwent senescence in response to irradiation and examined for molecular markers of the senescent phenotype. As shown in Figure 2A, STS93 and STS109 cells developed SA-β-gal activity after exposure to 2 Gy. The proportion of cells that appeared morphologically larger with SA-β-gal activity increased at 4 Gy, and nearly all cells were positive following 10 Gy of radiation. In contrast, STS117 cells did not develop SA-β-gal activity until exposed to 10 Gy of radiation, and only a very small proportion of cells were positive for staining. EdU incorporation, a measure of DNA synthesis and proliferative ability, revealed reduced DNA synthesis in the cell lines at different levels. We observed an almost complete disappearance of DNA synthesis in STS93 and STS109 while 50% of STS117 cells were EdU positive following the 24 h pulse interval from day 5 (Figure 2B).

Senescence phenotypes could be induced by the accumulation of unresolved DNA damage which may have resulted from irradiation. If so, those cells should also present a senescence-associated secretory phenotype (SASP) that is at least in part activated by DNA damage response signaling [24,25,27,28]. First, we used immunofluorescence to stain for DNA-damage associated γH2AX and 53BP1 foci and observed a large number of DNA damage foci that persisted for 10 days following treatment and detected genomic instability from the presence of micronuclei [46] (Figure 2C). This was consistent with the induction at the mRNA level of Interleukin-6 (IL-6) and Interleukin-8 (IL-8), two well-described cytokines of the SASP. After exposure to 8 Gy, we measured a strong and progressive increase of both IL-6 and IL-8 over time in STS109 and STS117, while STS93 showed no significant induction of IL-6 or IL-8 at the RNA level (Figure 2D). When cytokine secretions of IL-6 and IL-8 were detected in the supernatant, all three cell lines showed an increased IL-8 secretion from irradiated condition, whereas IL-6 secretion was detected only for STS109 and STS117 (Figure 2E). Combining these results, we demonstrated that treatment with RT induced multiple senescence hallmarks in STS cells suggesting a senescence status that may be targeted to potentiate cell death.

Senescence phenotypes could be induced by the accumulation of unresolved DNA damage which may have resulted from irradiation. If so, those cells should also present a senescence-associated secretory phenotype (SASP) that is at least in part activated by DNA damage response signaling [24,25,27,28]. First, we used immunofluorescence to stain for DNA-damage associated γH2AX and 53BP1 foci and observed a large number of DNA damage foci that persisted for 10 days following treatment and detected genomic instability from the presence of micronuclei [46] (Figure 2C). This was consistent with the induction at the mRNA level of Interleukin-6 (IL-6) and Interleukin-8 (IL-8), two well-described cytokines of the SASP. After exposure to 8 Gy, we measured a strong and progressive increase of both IL-6 and IL-8 over time in STS109 and STS117, while STS93 showed no significant induction of IL-6 or IL-8 at the RNA level (Figure 2D). When cytokine secretions of IL-6 and IL-8 were detected in the supernatant, all three cell lines showed an increased IL-8 secretion from irradiated condition, whereas IL-6 secretion was only detected for STS109 and STS117 (Figure 2E). Combining these results, we demonstrated that treatment with RT induced multiple senescence hallmarks in STS cells suggesting a senescence status that may be targeted to potentiate cell death.

### 2.3. RT Modulates Bcl-2 Family of Anti-Apoptotic Proteins

**Figure 3:**
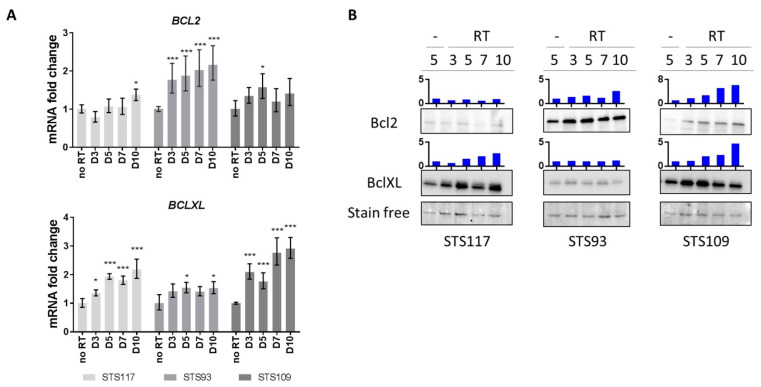
Anti-apoptotic proteins of the Bcl-2 family are upregulated by radiation. (**A**) Relative mRNA levels of BCL-2 and BCL-XL over time evaluated by real-time qPCR following 8 Gy of radiation. The values represent fold change expression relative to untreated controls. (**B**) Western blot analysis of BCL-2 and BCL-XL protein levels of untreated control (-) at Day 5 and irradiated (RT) cells over time. Stain free is a representative band of total protein acquired by stain-free technology. Bar graph (in blue) represent the protein quantification relative to the total protein. Data in (**A**) were analyzed using two-tail Student’s t-test to compare untreated vs. RT treated cells. * *p* < 0.05, *** *p* < 0.001. Data representative of three experiments for qPCR and one experiment for western blot. Error bars indicate ± the standard deviation.

During senescence, the Bcl-2 family of anti-apoptotic proteins is essential in order for cells to resist apoptosis and maintain survival [37,39,47,48]. The effect of RT on BCL-XL and BCL-2 expression levels was measured in our STS cells. At the mRNA level, BCL-XL increased significantly in STS109 and STS117, but not in STS93 during senescence establishment (Figure 3A). On the other hand, only STS93 presented a persistent increase in BCL-2 gene expression level over time. This was consistent with protein levels, with STS109 and STS117 showing an increased BCL-XL level over time in addition to present higher basal levels than STS93 (Figure 3B and Figure S1). While BCL2 expression is stronger in STS93, proteins analysis revealed an increase in both STS93 and STS109 with RT. Accordingly, the cell lines presented a different anti-apoptotic expression profile and modulation of mRNA and protein levels visible from day 3 were maintained 10 days after irradiation. This reinforced the hypothesis of a senescent state in RT treated STS cells, with each cell lines presenting different patterns of TIS hallmarks.

### 2.4. Targeting Bcl-2 Family Proteins Specifically Eliminates Irradiated Sarcoma Cells

Drugs that target the Bcl-2 family of anti-apoptotic proteins have been described as senolytic agents that can kill senescent cells [37,38,48]. We selected two commonly used BH3-mimetics: ABT-263 (Navitoclax) targets BCL-2/BCL-XL/BCL-W [49] and ABT-199 (Venetoclax) targets only BCL2 but with greater affinity [50]. ABT-263 or ABT-199 was applied to untreated or pre-irradiated cells. For the latter condition, cells were first exposed to 8 Gy of radiation and incubated for 5 days before the addition of drugs. While some senescence biomarkers appeared later in time, like SA-β-gal activity, the upregulation of Bcl-2 family members shown in Figure 3A,B suggests that 5 days post-RT is sufficient to target Bcl-2 family proteins. This was also consistent with the post-treatment time window that other groups used to demonstrate the effect of senolytic on TIS cells [37,38,39,42].

**Figure 4:**
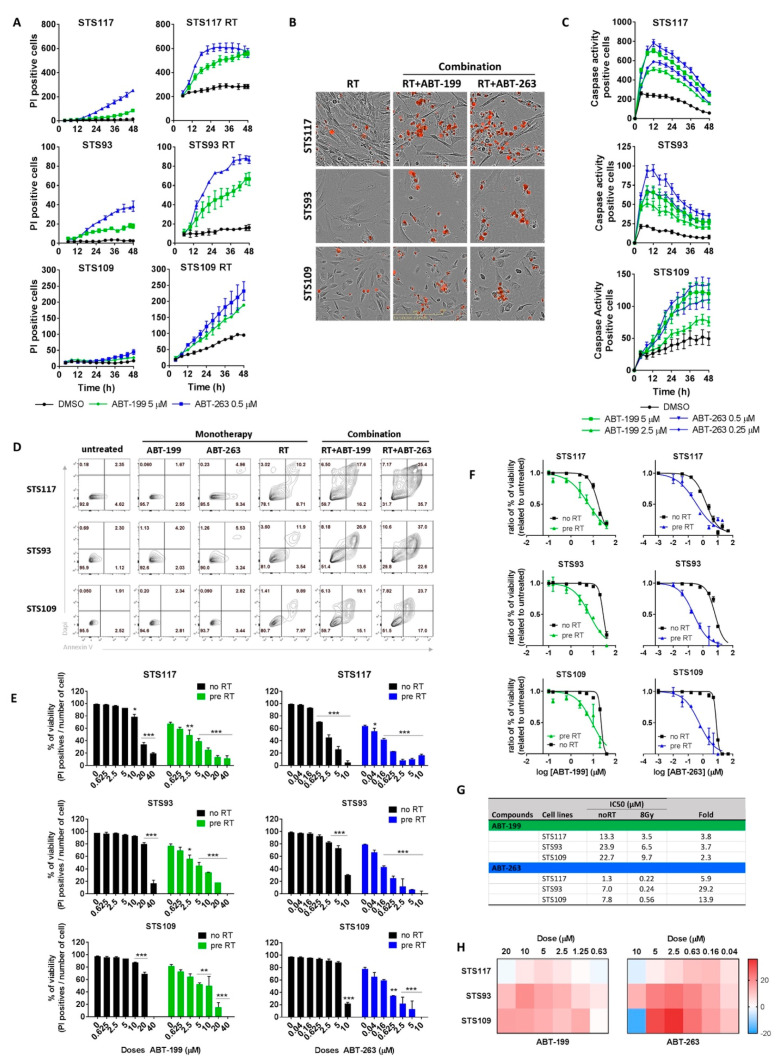
ABT-263 or ABT-199 induces rapid and specific cell death in irradiated sarcoma cells. (**A**) Real-time cell death curve of propidium iodide (PI) incorporation in untreated (left) or pre-irradiated (RT; 8 Gy, 5 days before) (right) sarcoma cell lines treated with ABT-263 (0.5 μM) or ABT-199 (5 μM). (**B**) Representative images of PI staining in irradiated cells treated with vehicle, ABT-263 or ABT-199. (**C**) Caspase 3-7 activity in pre-irradiated cells (8 Gy) treated with ABT-263 (0.25 and 0.5 μM) or ABT-199 (2.5 and 5 μM). (**D**) Flow cytometry analysis of apoptosis in untreated cells, cells treated with ABT-199 (10 μM) or ABT-263 (1 μM) or RT alone (8 Gy) (monotherapy), and pre-irradiated cells treated with either senolytic in combination. (**E**) Cytotoxicity evaluated by percentage of PI positive cells over total number of cells (Cell viability (%)) in control or pre-irradiated cells (8 Gy) 48 h after treatment with increasing doses of ABT-199 or ABT-263. (**F**) Doses response curves for ATB-199 and ABT-263 treatment of RT (8 Gy) or untreated cells for in each cell lines evaluated from % of viability relative to DMSO exposed cells. (**G**) IC50 values for both compounds. (**H**) Heat map of Excess over bliss for combination treatments of radiation and different concentrations of ABT-263 and ABT-199. Data in (**E**) were analyzed using ANOVA for multiple comparison with the vehicle treated control. * *p* < 0.05, ** *p* < 0.01, *** *p* < 0.001. Data are representative of three experiments and the Bliss score data are the mean of three independent experiments. Error bars indicate ± the standard deviation.

Using live-cell imaging, we followed the cytotoxic effects of different drug combinations over time. When added to non-irradiated control cells, ABT-199 or ABT-263 as a monotherapy failed to induce any substantial cell death in the cell lines, as measured by propidium iodide (PI) incorporation (Figure 4A, left panel). In contrast, when cells were pre-irradiated to induce a senescence-like phenotype, the same treatment resulted in a rapid accumulation of dead cells (Figure 4A,B). In pre-irradiated STS117 and STS93 cells, cell death was detected immediately after adding either drug. Although STS109 responded to both drugs, the accumulation of dead cells was more gradual (Figure 4A).

To confirm that ABT-199 and ABT-263 reduced cell viability through induction of apoptosis, we used a fluorescent reporter of caspases 3 and 7 activity in our cell lines in real-time imaging. Compared to cells treated with RT alone, we detected a rapid increase of caspase activity in cells that received the combination of radiation with either senolytics and reached a plateau after 12 h for STS93 and STS117 (Figure 4C). In agreement with these results, cell death measured by a more conventional assay of flow cytometry also showed specific cell killing of irradiated cells by Bcl2 family of anti-apoptotic inhibitors even at higher drug concentrations (1 µM of ABT-263 and 10 µM of ABT-199) (Figure 4D). After 48 h of treatment, Figure 4D shows that inhibitors or RT used as monotherapy had a modest effect on STS cell survival, whereas the combination of RT with inhibitors resulted in the rapid detection of dead cells.

When examining drug sensitivity across a larger range of concentrations, dose-dependent therapeutic effect on viability was observed with both inhibitors when cells where previously irradiated (Figure 4E). Without RT, high doses of ABT-199 and ABT-263 are needed to induce STS cell death, suggesting that Bcl-2 family inhibitors are not effective as monotherapy (Figure 4E). In a study from Teicher et al. [51], sarcoma cell lines exposure to ABT-199 or ABT-263 also revealed resistance of this pathology to these compounds. Importantly, BCL-2 selective inhibitor ABT-199 improved cell death in irradiated STS117 and STS109, despite their low levels of BCL-2 and the upregulation of expression of BCL-XL (Figure 3A,B) suggesting that BCL-2 may be a central mediator of STS apoptosis resistance to RT. However, IC50 of ABT-199 were approximately twenty times higher than ABT-263 (Figure 4F,G), which supported the need for a BCL-XL and/or BCL-W-selective counterpart to recapitulate ABT-263 efficacy. Leverson et al. obtained the same result with these molecules on an extended cancer cell lines panel [52]. According to IC50′s, STS117 was the most sensitive cell line for both drugs and STS109, as already mentioned, presents a different kinetic which was attested by higher IC50 for both drugs (Figure 4F,G). For this cell line, when IC50 was determined later in time (at 72 h), an IC50 of 6.2 µM could be reached. Using bliss independent model [53], we demonstrated that combination of 8 Gy of radiation and senolytics was synergistic. Synergistic killing was observed in all cell lines with concentration from 1.25 µM to 10 µM for ABT-199 and 0.04 to 5 µM for ABT-263 (Figure 4H).

We conclude that RT, by inducing DNA-damage followed by an increase in expression of BCL-2 family of anti-apoptotic proteins, sensitized STS cells to ABT-199 and ABT-263. From these results, we demonstrated for the first time that the combination of RT and senolytics can efficiently alter the fate of irradiated sarcoma cells from senescence to apoptosis.

### 2.5. ABT-263 and ABT-199 Enhance Radiation Toxicity in a Three-Dimensional Model

**Figure 5:**
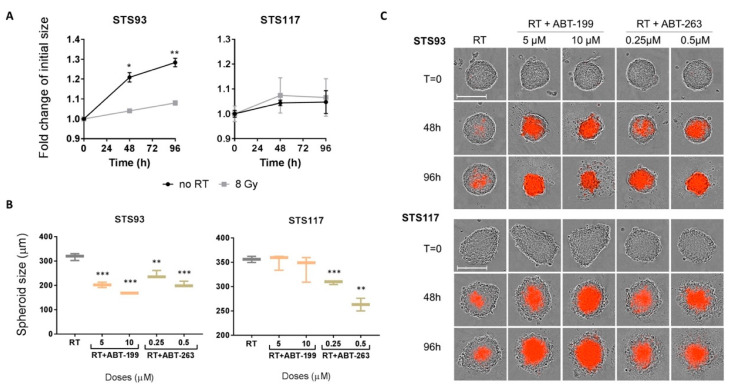
Senolytics can induced cell death in irradiated 3D model of sarcoma cells. (**A**) Fold change in size over time of spheroids untreated or exposed to 8 Gy. (**B**) Size of spheroids irradiated (RT; 8 Gy) or combination of RT and ABT-263 (0.25 μM and 0.5 μM) or ABT-199 (5 μM and 10 μM). Measurements represent size 96 h after treatment. (**C**) Representative pictures of PI incorporation in irradiated spheroids treated with combination treatment over time. T = 0 represents cells prior to drug addition. The scale bar represents 300 μM. Data from (**A**) and (**B**) were analyzed using the two-tail Student’s t-test to compare treatment groups with the control group. * *p* < 0.05, ** *p* < 0.01, *** *p* < 0.001. Data are representative of three experiments. Error bars indicate ± the standard deviation.

To further evaluate the combination of RT with senolytic drugs, we used a three-dimensional (3D) STS spheroid model, which may better recapitulate in vivo tumour response [54]. Both STS93 and STS117 formed suitable spheroid structures, whereas STS109 cells did not aggregate and could not be tested using 3D models. Untreated STS93 spheroids increased in size with time, reflecting the viability of cells cultured as spheroids, while STS117 remained the same in size over 96 h (Figure 5A). We propose that within this short period of time, irregular STS117 spheroids became more compact, which could explain the absence of increase in size. When treated with RT alone, spheroid sizes remained constant suggesting a partial response to RT with blunted proliferation and absence of immediate cell death (Figure 5A). This result was consistent with the lack of clinical tumour size regression following pre-operative RT in most STS subtypes.

As expected, when a single dose of ABT-263 or ABT-199 was added to pre-irradiated STS93 grown in 3D, spheroids shrunk significantly compared to those treated with RT alone. STS117 spheroids responded more drastically to ABT-263 than ABT-199 according to their size (Figure 5B). However, despite a lack of spheroid size response, the presence of cell death in pre-irradiated spheroids treated with both ABT-199 or ABT-263 was confirmed with a strong increase in PI staining over time for both cell lines (Figure 5C). PI staining also indicated very little cell death with RT alone (Figure 5C). The discordance between 3D spheroid size of STS117 and its sensitivity to senolytic in 2D cultures may be due to the disaggregation of dead cells in spheroids leading to overestimated sizes following treatment. This cell-line dependent bias in area measurement has been reported previously by others [55]. Thus, from a 3D culture model, we concluded that ABT-263 produced a higher cytotoxic effect then ABT-199 by removing peripheral cell layers in STS117, while ABT-199 (5 µM) and ABT-263 (0.5 µM) presented an equivalent potency to kill pre-irradiated STS93. Overall, our results demonstrated that the combination of Bcl-2 inhibitors and RT efficiently enhances cytotoxicity and cell death.

## 3. Discussion

Pre-operative RT before surgery is commonly used in STS management. However, RT does not induce sufficient tumour volumetric response to impact surgery size or feasibility in most STS. Using UPS cell lines, one of the more common and aggressive STS subtypes [56], we observed that RT induced a senescence-like phenotype rather than cell death, which corroborates the lack of tumour size reduction in clinic in high-grade sarcomas refractory to RT [8,9,10,11]. We elaborated a new therapeutic strategy to enhance tumour response to pre-operative RT by targeting these senescent cells with Bcl-2 family inhibitors to induce apoptotic cell death.

**Figure 6:**
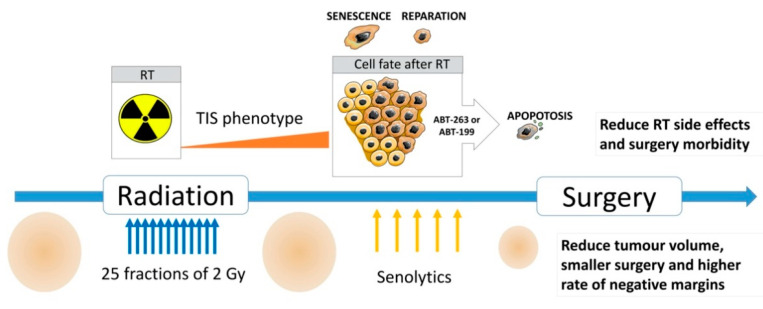
Potential clinical strategy for curative STS. Model in three steps proposed for management of treatment timeline of patient. For sarcomas patient, pre-operative radiation is given in several fractions and is followed by an interval of six to ten weeks before surgery. During this period of time, we hypothesize that the tumour will undergo radiation-induced senescence and this state will sensitize the cells for the use of senolytic drugs. This addition to conventional treatment may enhance tumour shrinkage and thus, potentially have positive effects on the success of the surgery as well as on reducing side effects cause by radiation over time.

In clinic, pre-operative RT of STS conventionally involves five weeks of RT followed by six to ten weeks of recovery that ends with curative or palliative surgery. The recovery interval between RT and surgery is necessary to reduce adverse complications related to RT, but also represents a window of opportunity to introduce senolytic therapy to boost the effects of RT without interfering with STS standard care (Figure 6). Results from STS may serve as a model for other cancers that include pre-operative RT in the management options, such as colorectal cancer [57], lung [58] and breast cancers [59].

ABT-199 is an FDA approved medication for use as monotherapy or in combination with other medications for the treatment of haematological malignancies. While phase I and II trials with ABT-199 for solid tumours are ongoing, they explore combination involving chemotherapies. In contrast to present clinical trials, our strategy aims to use geographically targeted RT to pre-condition tumours to alter their sensitivity to ABT-199. On the other hand, ABT-263 has also been administered for solid tumours [60]. Even though ABT-263 has been extensively used with success in preclinical models, its toxicity has restricted its use in clinic [60,61,62]. The thrombocytopenia associated with ABT-263 is linked to BCL-XL inhibition [63] and can be avoided with ABT-199 which is specific to BCL2 [50,64]. Even if ABT-263 has been proven to be more potent in our work (Figure 4) as well as in previous reports [40,42,52], ABT-199 seems to be more suitable for clinical administration. The concentrations of ABT-199 used here were much higher than those previously described in vitro for haematological malignancies ranging from nanomolar to 1 μM but could be relevant for solid tumours [50,65,66]. It should be noted that ABT-199 can achieve and maintain plasma exposures around 1–3 μM at daily doses ranging from 400 to 800 mg and that doses as high as 800 mg per day were not associated with serious toxicity in clinical study [67,68,69,70].

Whether the lowered IC50 from pre-irradiation render ABT-199 clinically effective remains to be tested, but the inhibition of multiple members of the Bcl-2 family will likely be needed to further reduce drug concentration and to address the diverse STS subtypes and tumour heterogeneity. In addition to ABT-199 and ABT-263, other Bcl-2 family inhibitors have been evaluated in clinical trials for cancer treatment [71], including molecules targeting MCL-1 (A-1210477, S63845, AMG 176, AZD5991) [72,73,74,75]. MCL-1 protein levels as well as BCL-XL have been demonstrated to influence sensitivity to ABT-199 and ABT-263 in other cancers types [73,76,77,78]. Notably, STS109 response to both ABT-199 and ABT-263 may indicate a partial resistance, reflected by a slower rate of cell death that may possibly rely on MCL-1 expression.

Differences in the senescence-associated phenotype between our cell lines were likely due to genomic differences. Among them, STS93 and STS109 carry wild type TP53 gene while STS117 carries mutated TP53 [43]. The complete senescence phenotype of STS93 and STS109 was in line with the senescence-associated growth arrest that generally relies on p53/p21 [21]. Still, senescence can be induced without p53 [42,79]. In the absence of strong senescence markers (Figure 2), we suspect that STS117 undergo mitotic catastrophe, which is the main cellular response to RT-induced DNA damage, especially in the absence of functional check points such as p53 [16,19,80] and can drive cells toward cell death by either apoptosis or necrosis or toward senescence. Given the low level of cell death in STS117 after RT (Figure 1 and Figure 4), mitotic slippage in STS117 likely resulted in senescence. Importantly, despite displaying a weaker senescent phenotype, we demonstrated that STS117 cells were still targetable by Bcl2-inhibitors to rapidly drive them toward an apoptotic cell death (Figure 4). Although UPS is one of the most common subtypes of STS [56] and the cell lines used in this work present different genetic and morphological features, there is a need to extend the analysis to more cell lines representing different subtypes of STS in the future. Further investigation will help to better predict tumour sensitivity to Bcl2-inhibitors. In vivo studies to validate this therapeutic strategy will also be done.

En-bloc resection of STS is an essential component in the curative treatment of the majority of patients. The feasibility and morbidity of surgery is in part associated with the size of the tumour. Approximately 13% of all newly diagnosed non-metastatic STS patients are not suitable to undergo surgery [81,82,83] partly due to potential surgical complications and anatomical closeness to critical organs. Reducing tumour size with senolytic treatment could ameliorate the surgeon’s ability to obtain a negative resection margin, reduce recurrences and surgical morbidity. Modern RT techniques such as intensity-modulated radiation therapy (IMRT) and stereotactic body radiation therapy (SBRT) facilitate the sparing of surrounding normal tissues and organs and reduce RT-related adverse effects [84]. Nevertheless, chronic RT side effects, such a fibrosis occur in up to 28% of long-term cancer survivors [85,86]. Thus, the administration of senolytics such as ABT-199 and ABT-263 may also reduce RT-induced fibrosis and organ dysfunctions, which is consistent with preclinical lung, liver, muscular and hematopoietic tissues models [36,37,40,41].

## 4. Materials and Methods

### 4.1. Cell Culture and Transduction

STS93, STS117 and STS109 are human STS cell lines that were provided by Dr R. Gladdy from The Lunenfeld-Tanenbaum Research Institute and previously described [43]. These cell lines were derived from biopsy or surgery of three patients diagnosed with stage IV UPS, prior to any adjuvant therapies. Cells were cultured in DMEM/F12 media supplemented with 10% fetal bovine serum and 1% penicillin-streptomycin. Cells were incubated at 37 °C with 5% CO2 and were used at a low passage number. To produce H2B-GFP expressing cell lines, lentiviruses were generated as previously described [42,87] and transduction was performed overnight in the presence of Polybrene. Cells were then selected for an appropriate period with hygromycin.

### 4.2. Irradiation and Senolytic Treatments

Ionizing radiation treatment was performed with the Gammacell 3000 (Best Theratronics, Ottawa, ON, Canada) at defined doses (0, 2, 4, 6, 8 or 10 Gy). We purchased ABT-199 (Venetoclax) that inhibited BCL2 or ABT-263 (Navitoclax) for BCL2/BCLxl/BclW from Medchem and APExBIO. Both drugs were prepared with Dimethyl sulfoxide (DMSO) and DMSO from Sigma was used for vehicle alone in control condition. In combination treatment, pre-irradiation conditions represented cells that were exposed to 8 Gy of radiation five days before adding drugs while control cells were seeded two to three days before drug addition.

### 4.3. Clonogenic Assay

Cells were irradiated, trypsinized then seeded onto petri dishes at various concentrations. After 7–14 days, colonies were fixed and stained in 20% methanol solution containing 0.5% crystal violet. Colonies were counted and surviving fractions were calculated by normalizing counts to the number of colonies of non-irradiated control cells of each cell line.

### 4.4. Cell Death and Cell Cycle Analysis by Flow Cytometry

Fluorescent activated cell sorting (FACS) was used to measure the amount of apoptotic, necrotic and living cells, and cell cycle stage. Cells were grown in a 6-well plate and maintained below 80% confluence at time of harvest. Following appropriated treatment time (see figure legends for treatment timing and sequence), media and cells were collected and washed with PBS. For cell death, cells were stained for 15 min at room temperature with Annexin V-AlexaFluor488 and then DAPI (1 µg/mL), all in Annexin V binding buffer. For cell cycle distribution, cells were fixed with 70% ethanol at 48 h after irradiation, washed with PBS, treated with RNase (100 ug/mL) and stained with propidium iodide (PI; 10 µg/mL) then sorted. FACS was performed using the LSR Fortessa cell analyzer (BD Bioscience, Mississauga, ON, Canada) and analyzed using the FlowJO software (Tree Star, Ashland, OR, USA).

### 4.5. Real-Time Imaging for Proliferation

For growth curve analysis, cells expressing H2B-GFP were treated with 0, 2, 6 or 8 Gy of radiation in a Gammacell 3000 (Best Theratronics) and seeded in a 96-well plate. Cell proliferation was followed using the IncuCyte ZOOM or IncuCyte S3 Live-cell Imaging System (Sartorius, Göttingen, Germany) using a 10× objective. The number of cells was evaluated using Incucyte^®^ Software by the number of green fluorescent nucleus of cells expressing H2B-GFP in at least two wells per condition. Experiments were performed at least three times.

### 4.6. Senescence-Associated β-Galactosidase Activity

SA-b-gal was assayed 10 days after irradiation (0, 2, 4 and 10 Gy) according to previously describe protocol [22]. Cells were fixed with 5% formalin for 5 min, wash with PBS and stained with fresh β-galactosidase staining solution (1 mg/mL 5-bromo-4-chloro-3-inolyl-β-galactosidase in dimethylformamide (20 mg/mL stock), 5 mM potassium ferricyanide, 150 mM NaCl, 40 mM citric acid/sodium phosphate and 2 mM MgCl_2_, at pH 6.0). Cells were incubated overnight at 37 °C and bright-field pictures of the cells were taken with EVOS microscope (Life Technologies, Burlington, ON, CA).

### 4.7. EdU (5-Ethynyl-2′-Deoxyuridine) Detection

For DNA synthesis detection, cells were treated with radiation (0 to 8 Gy) and incubated for 5 days. EdU was added to the medium and incubated for 24 h prior fixation. After this pulse, cells were washed with PBS and fixed with 10% formalin for 10 min. EdU staining was assessed using the Click-iT™ EdU Alexa Fluor 647™ Imaging Kit (Invitrogen, Burlington, ON, CA). SYTOX™ Green (Invitrogen) staining were used for total number of cell count. Images were obtained using IncuCyte S3 Live-cell Imaging System and analyzed using Incucyte^®^ Software (Version 2020B, Sartorius, Göttingen, Germany).

### 4.8. DNA-Damage Detection

After irradiation, cells were plated in chamber slides and fixed 10 days later with 10% formalin for 5 min. Non-irradiated controls were seeded 3 days before fixation. Cells were permeabilized with 0.25% Triton for 10 min, incubated in blocking solution (4% donkey serum, 1% BSA, PBS) for 1 h and then incubated overnight at 4 °C with primary antibodies against γ-H2AX (which designates the phosphorylated form of H2AX at Ser139) (1:2000 dilution; JBW301) and 53BP1 (1:2000, clone 305) (Millipore and Novus respectively). Cells were washed and incubated with secondary antibody Alexa fluor-564 or Alexa fluor-647 (1:750) (Invitrogen) for 1 h at room temperature and then washed again. Prolong containing DAPI was used for slide mounting and images were obtained using a Zeiss Observer Z1 microscope (400×).

### 4.9. Quantitative Real-Time PCR

RNA was isolated from cells using the Total RNA Purification Plus Kit from NORGEN Biotek Corp (Thorold, ON, Canada). Briefly, cells were washed with PBS and lysis buffer was applied directly to plates. Extraction was performed following the manufacturer’s instruction. RNA concentrations and quality were evaluated using a Nanodrop 1000 (Thermo Fisher Scientific, Saint-Laurent, QC, Canada). RNA was reversed transcribed using the QuantiTect Reverse Transcription Kit (Qiagen Inc., Toronto, ON, Canada). Q-PCR was performed using sequence specific primers and the SYBR Greener Mix (Invitrogen). Sequence primers for target genes IL-6, IL-8, BCL2 and BCLXL are described in the “List of primers” section. Q-PCR was performed using Applied BioSystems^®^ (Saint-Laurent, QC, Canada) QuantStudio 7 Flex apparatus. Gene expression values were normalized to both TATA-binding protein gene expression (TBP) and Hypoxanthine Phosphoribosyltransferase 1 (HRPT). The Pfaffl analysis method (Pffl 2001) was applied to data generated by Q-PCR.

### 4.10. List of Primers

IL-6: 5′-TGTGTGAAAGCAGCAAAGA-3′ F; 5′-GGCAAGTCTCCTCATTGAA-3′ R

IL-8: 5′-GCCAACACAGAAATTATTGTAAAG-3′ F; 5′-TTATGAATTCTCAGCCCTCTTC-3′ R

BCL-2: 5′AACATCGCCCTGTGGATGAC-3′ F; 5′GGCCGTACAGTTCCACAAAG-3′ R

BCL-XL: 5′GGCCACTTACCTGAATGACC-3′ F; 5′AAGAGTGAGCCCAGCAGAAC-3′ R

TBP: 5′-CCACTCACAGACTCTCACAAC-3′ F; 5′-CTGCGGTACAATCCCAGAACT-3′ R

HPRT: 5′-CCTGGCGTCGTGATTAGTGAT-3′ F; 5′-AGACGTTCAGTCCTGTCCATA-3′ R

### 4.11. Western Blot Analysis

For whole proteins extraction, M-PER protein extraction reagent (Thermo Scientific, Saint-Laurent, QC, Canada)) with Complete inhibitor cocktail (Roche, Laval, QC, Canada) were applied to cell pellets. Protein concentration was measured using Nanodrop 1000 (Thermo Scientific, Saint-Laurent, QC, Canada). Cell lysates were loaded onto 4-15% Mini PROTEAN^®^ TGX Stain-Free™ Gels (Bio-Rad, Saint-Laurent, QC, Canada) and then transferred onto PVDF membrane. PBS solution containing 5% BSA was used for blocking and antibody incubation. Antibodies used in western blotting include BCL2 (C124), BCLXL (clone 54H6) and HRP-coupled secondary. The control of the loading protein was evaluated using the stain-free technology (Bio-Rad Laboratories, Saint-Laurent, QC, Canada). Chemiluminescence was detected using the ChemiDoc MP Imaging System (Bio-Rad Laboratories, Saint-Laurent, QC, Canada). Quantification of the relative signal intensity of the protein bands were obtained using the ratio of the protein bands over the corresponding complete stain-free lane. The ratios were then normalized to the control. Computation was performed using a homemade code with Mathematica 12 (Wolfram, Champaign, IL, USA).

### 4.12. Cytokine Secretion Measurement

Conditioned media for untreated or irradiated cells were prepared ten days after irradiation or 36 h after seeding for untreated condition, cells were washed and incubated in medium without FBS for 24 h. Levels of IL-6 and IL-8 were assessed using multisport electrochemiluminescence immunoassay system using the V-Plex human kit from Meso Scale Discovery (MSD #K15209D). The data were normalized according to cell counts (pg/mL/10^6^ cells).

### 4.13. Real-Time Imaging for Cell Death and Apoptosis

For drug treatment, 50 µL of fresh media containing three times the desired concentration of ABT-199, ABT-263, DMSO or media only were added to the 100 µl of media in 96-well plates. Cell death and Caspase activity were measured with PI (1 µg/mL) or CellEvent Caspase 3/7 Green (3 drops per ml, according to manufacturer protocol). Reagents were added to the prepared drug solution. Cells were followed using the IncuCyte ZOOM or IncuCyte S3 Live-cell Imaging System (Sartorius, Göttingen, Germany) using a 10× objective. The percentage of cell survival was calculated using H2B-expressing cells according to the formula: number of PI positive cells over the number of cells (H2B-GFP nucleus). For apoptosis assessment, number of green objects was calculated by software. Experiments were performed at least three times.

### 4.14. Drug Combination Analysis

Using real-time imaging system with H2B-GFP expressing cells and PI staining, the percentage of viability was defined three days after drug treatment from (total number of cells—PI positive cells), normalize to day 0. Combination activity was assessed using the Bliss independent model [53], with negative values indicate antagonism, values around zero indicate additive effects and positive values indicate synergy. Bliss scores were calculated for each combination of the dose matrix (1 × 6, one dose of radiation and 6 doses of ABT-263 or ABT-199). Excess over bliss score is shown in the figure and represents the mean of three independent experiments.

### 4.15. 3D Cell Culture

To form spheroids from STS93 and STS117, 2500 cells were seeded in an ultra-low attachment 96-well plates (Corning Life Sciences, Tewksbury, MA, USA). Cells were incubated for at least two days to allow the formation of spheroids and then treated with radiation (8 Gy). Three days later ABT-263 (0.25 and 0.5 μM), ABT-199 (5 and 10 μM) or DMSO as a control was added. Spheroid size was monitored over time and evaluated from the mean of two measurements/spheroid, three spheroids per condition.

### 4.16. Statistical Analysis

Data were analyzed for significance using Student’s t-test and one-way analysis of variance (ANOVA) using GraphPad Prism software (Version 7, GaphPad, San Diego, CA, USA) or MS Excel Office 365 (Version 15.0, Microsoft Canada, Montreal, QC, Canada). IC50 were calculated with Graph Pad Prism software.

## 5. Conclusions

The senescence-like phenotype observed after RT treatment of UPS cell lines reflects the lack of important tumour size reduction in most subtypes of STS following pre-operative RT. We hypothesize that pre-operative RT-induced senescence in STS can be exploited to modify cell fate from senescence towards a rapid cell death through the administration of a Bcl2 family anti-apoptotic proteins inhibitor in the time window between RT and surgery. Important reduction in tumour size will facilitate STS surgery and minimize associated morbidities to improve patient outcomes. Moreover, this strategy may be especially beneficial for more radio-resistant subtypes of STS in the future. A recent clinical trial suggested the de-escalation in the dose of RT in the pre-operative treatment of radiosensitive myxoid liposarcoma is safe and does not impair local control [88]. Perhaps, targeting RT-resistant STS using our strategy will ultimately lead to similar volumetric and dosimetric results as observed in myxoid liposarcomas. Further investigation will be needed to find good predictors of both RT sensitivity and responsiveness to BCL2-inhibitors in patients to ultimately define which inhibitor will be more potent and safer for them.

## Data Availability

The data presented in this study are available on request from the corresponding author.

## Supplementary Materials

The following are available online at https://www.mdpi.com/2072-6694/13/3/386/s1, Figure S1: Western blot and densitometry analysis of BCL2 and BCL-XL.

## Abbreviations

The following abbreviations are used in this manuscript:

53BP1	Tumor suppressor p53-binding protein 1
Bcl-2	B-cell lymphoma 2
Bcl-xL	B-cell lymphoma-extra large
BH3	Bcl-2 homology domain 3
BSA	Bovine serum albumin
DAPI	4′,6-diamidino-2-phenylindole fluorescent stain
DMEM	Dulbecco’s modified eagle medium
DMSO	Dimethyl sulfoxide
EdU	5-Ethynyl-2’-deoxyuridine
FACS	Fluorescent activated cell sorting
FBS	Fetal bovine serum
FDA	Food and drug administration
GFP	Green fluorescent protein
H2B	Histone H2B
HRPT	Hypoxanthine Phosphoribosyltransferase 1
HRP	Horseradish peroxidase
IL-6	Interleukin 6
IL-8	Interleukin 8
IMRT	Intensity-modulated radiation therapy
LR	Local recurrence
MCL-1	Induced myeloid leukemia cell differentiation protein
PARP	Poly (ADP-ribose) polymerase
PBS	Phosphate-buffered saline
PI	Propidium iodide
PVDF	Polyvinylidene fluoride
RT	Radiation therapy
SASP	Senescence associated secretory phenotype
SA-β-gal	Senescence associated beta-galactosidase
SBRT	Stereotactic body radiation therapy
STS	Soft-tissue sarcoma
TBP	TATA-binding protein gene expression
TIS	Therapy-induced senescence
UPS	Undifferentiated pleomorphic sarcoma
γH2AX	Phosphorylated H2AX (Ser139)

## References

[1] Canadian Cancer Society and National Cancer Institute of Canada, Advisory Committee on Records and Registries (2019). Canadian Canacer Statistics.

[2] Beane J.D., Yang J.C., White D., Steinberg S.M., Rosenberg S.A., Rudloff U. (2014). Efficacy of Adjuvant Radiation Therapy in the Treatment of Soft Tissue Sarcoma of the Extremity: 20-year Follow-Up of a Randomized Prospective Trial. Ann. Surg. Oncol..

[3] O’Sullivan B., Davis A.M., Turcotte R., Bell R., Catton C., Chabot P., Wunder J., Kandel R., Goddard K., Sadura A. (2002). Preoperative versus postoperative radiotherapy in soft-tissue sarcoma of the limbs: A randomised trial. Lancet.

[4] Rosenberg S.A., Tepper J., Glatstein E., Costa J., Baker A., Brennan M., DeMoss E.V., Seipp C., Sindelar W.F., Sugarbaker P. (1982). The treatment of soft-tissue sarcomas of the extremities: Prospective randomized evaluations of (1) limb-sparing surgery plus radiation therapy compared with amputation and (2) the role of adjuvant chemotherapy. Ann. Surg..

[5] Davis A.M., O’Sullivan B., Turcotte R., Bell R., Catton C., Chabot P., Wunder J., Hammond A., Benk V., Kandel R. (2005). Late radiation morbidity following randomization to preoperative versus postoperative radiotherapy in extremity soft tissue sarcoma. Radiother. Oncol..

[6] Diamantis A., Baloyiannis I., Magouliotis D.E., Tolia M., Symeonidis D., Bompou E., Polymeneas G., Tepetes K. (2020). Perioperative radiotherapy versus surgery alone for retroperitoneal sarcomas: A systematic review and meta-analysis. Radiol. Oncol..

[7] Greto D., Livi L., Saieva C., Bonomo P., Meattini I., Loi M., Di Brina L., Beltrami G., Campanacci D., Scoccianti G. (2013). Neoadjuvant treatment of soft tissue sarcoma. Radiol. Med..

[8] Wong P., Dickie C., Lee D., Chung P., O’Sullivan B., Letourneau D., Xu W., Swallow C., Gladdy R., Catton C. (2014). Spatial and volumetric changes of retroperitoneal sarcomas during pre-operative radiotherapy. Radiother. Oncol..

[9] Canter R.J., Martinez S.R., Tamurian R.M., Wilton M., Li C.S., Ryu J., Mak W., Monsky W.L., Borys D. (2010). Radiographic and histologic response to neoadjuvant radiotherapy in patients with soft tissue sarcoma. Ann. Surg. Oncol..

[10] Gui C., Morris C.D., Meyer C.F., Levin A.S., Frassica D.A., Deville C., Terezakis S.A. (2019). Characterization and predictive value of volume changes of extremity and pelvis soft tissue sarcomas during radiation therapy prior to definitive wide excision. Radiat. Oncol. J..

[11] Roberge D., Skamene T., Nahal A., Turcotte R.E., Powell T., Freeman C. (2010). Radiological and pathological response following pre-operative radiotherapy for soft-tissue sarcoma. Radiother. Oncol..

[12] Lauber K., Ernst A., Orth M., Herrmann M., Belka C. (2012). Dying cell clearance and its impact on the outcome of tumor radiotherapy. Front. Oncol..

[13] Wong P., Houghton P., Kirsch D.G., Finkelstein S.E., Monjazeb A.M., Xu-Welliver M., Dicker A.P., Ahmed M., Vikram B., Teicher B.A. (2014). Combining Targeted Agents With Modern Radiotherapy in Soft Tissue Sarcomas. J. Natl. Cancer Inst..

[14] Rebbaa A., Zheng X., Chou P.M., Mirkin B.L. (2003). Caspase inhibition switches doxorubicin-induced apoptosis to senescence. Oncogene.

[15] Chang B.D., Broude E.V., Dokmanovic M., Zhu H., Ruth A., Xuan Y., Kandel E.S., Lausch E., Christov K., Roninson I.B. (1999). A senescence-like phenotype distinguishes tumor cells that undergo terminal proliferation arrest after exposure to anticancer agents. Cancer Res..

[16] Eriksson D., Stigbrand T. (2010). Radiation-induced cell death mechanisms. Tumor Biol..

[17] Te Poele R.H., Okorokov A.L., Jardine L., Cummings J., Joel S.P. (2002). DNA damage is able to induce senescence in tumor cells in vitro and in vivo. Cancer Res..

[18] Gewirtz D.A., Holt S.E., Elmore L.W. (2008). Accelerated senescence: An emerging role in tumor cell response to chemotherapy and radiation. Biochem. Pharmacol..

[19] Patel N.H., Sohal S.S., Manjili M.H., Harrell J.C., Gewirtz D.A. (2020). The Roles of Autophagy and Senescence in the Tumor Cell Response to Radiation. Radiat. Res..

[20] Di Fagagna F.D.A., Reaper P.M., Clay-Farrace L., Fiegler H., Carr P., Von Zglinicki T., Saretzki G., Carter N.P., Jackson S.P. (2003). A DNA damage checkpoint response in telomere-initiated senescence. Nature.

[21] Beauséjour C.M., Krtolica A., Galimi F., Narita M., Lowe S.W., Yaswen P., Campisi J. (2003). Reversal of human cellular senescence: Roles of the p53 and p16 pathways. EMBO J..

[22] Dimri G.P., Lee X., Basile G., Acosta M., Scott G., Roskelley C., Medrano E.E., Linskens M., Rubelj I., Pereira-Smith O. (1995). A biomarker that identifies senescent human cells in culture and in aging skin in vivo. Proc. Natl. Acad. Sci. USA.

[23] Itahana K., Campisi J., Dimri G.P. (2007). Methods to detect biomarkers of cellular senescence: The senescence-associated beta-galactosidase assay. Methods Mol. Biol..

[24] Rodier F., Coppé J.-P., Patil C.K., Hoeijmakers W.A.M., Muñoz D.P., Raza S.R., Freund A., Campeau E., Davalos A.R., Campisi J. (2009). Persistent DNA damage signalling triggers senescence-associated inflammatory cytokine secretion. Nat. Cell Biol..

[25] Rodier F., Muñoz D.P., Teachenor R., Chu V., Le O., Bhaumik D., Coppé J.-P., Campeau E., Beauséjour C.M., Kim S.-H. (2010). DNA-SCARS: Distinct nuclear structures that sustain damage-induced senescence growth arrest and inflammatory cytokine secretion. J. Cell Sci..

[26] Acosta J.C., O’Loghlen A., Banito A., Guijarro M.V., Augert A., Raguz S., Fumagalli M., Da Costa M., Brown C., Popov N. (2008). Chemokine Signaling via the CXCR2 Receptor Reinforces Senescence. Cell.

[27] Kuilman T., Michaloglou C., Vredeveld L.C., Douma S., Van Doorn R., Desmet C.J., Aarden L.A., Mooi W.J., Peeper D.S. (2008). Oncogene-Induced Senescence Relayed by an Interleukin-Dependent Inflammatory Network. Cell.

[28] Coppé J.-P., Patil C.K., Rodier F., Sun Y., Muñoz D.P., Goldstein J.N., Nelson P.S., Desprez P.-Y., Campisi J. (2008). Senescence-Associated Secretory Phenotypes Reveal Cell-Nonautonomous Functions of Oncogenic RAS and the p53 Tumor Suppressor. PLoS Biol..

[29] Hernandez-Segura A., Nehme J., DeMaria M. (2018). Hallmarks of Cellular Senescence. Trends Cell Biol..

[30] Wang E. (1995). Senescent human fibroblasts resist programmed cell death, and failure to suppress bcl2 is involved. Cancer Res..

[31] Soto-Gamez A., Quax W.J., DeMaria M. (2019). Regulation of Survival Networks in Senescent Cells: From Mechanisms to Interventions. J. Mol. Biol..

[32] Rea I.M., Gibson D.S., McGilligan V., McNerlan S.E., Alexander H.D., Ross O.A. (2018). Age and Age-Related Diseases: Role of Inflammation Triggers and Cytokines. Front. Immunol..

[33] Milanovic M., Fan D.N.Y., Belenki D., Däbritz J.H.M., Zhao Z., Yu Y., Dörr J.R., Dimitrova L., Lenze D., Barbosa I.A.M. (2018). Senescence-associated reprogramming promotes cancer stemness. Nat. Cell Biol..

[34] Lee S., Schmitt C.A. (2019). The dynamic nature of senescence in cancer. Nat. Cell Biol..

[35] Ruhland M.K., Loza A.J., Capietto A.-H., Luo X., Knolhoff B.L., Flanagan K.C., Belt B.A., Alspach E., Leahy K., Luo J. (2016). Stromal senescence establishes an immunosuppressive microenvironment that drives tumorigenesis. Nat. Commun..

[36] Zhu Y., Tchkonia T., Pirtskhalava T., Gower A.C., Ding H., Giorgadze N., Palmer A.K., Ikeno Y., Hubbard G.B., Lenburg M.E. (2015). The Achilles’ heel of senescent cells: From transcriptome to senolytic drugs. Aging Cell.

[37] Chang J., Wang Y., Shao L., Laberge R.-M., DeMaria M., Campisi J., Janakiraman K., Sharpless N.E., Ding S., Feng W. (2016). Clearance of senescent cells by ABT263 rejuvenates aged hematopoietic stem cells in mice. Nat. Med..

[38] Wang L., De Oliveira R.L., Wang C., Neto J.M.F., Mainardi S., Evers B., Lieftink C., Morris B., Jochems F., Willemsen L. (2017). High-Throughput Functional Genetic and Compound Screens Identify Targets for Senescence Induction in Cancer. Cell Rep..

[39] Yosef R., Pilpel N., Tokarsky-Amiel R., Biran A., Ovadya Y., Cohen S., Vadai E., Dassa L., Shahar E., Condiotti R. (2016). Directed elimination of senescent cells by inhibition of BCL-W and BCL-XL. Nat. Commun..

[40] Pan J., Li D., Xu Y., Zhang J., Wang Y., Chen M., Lin S., Huang L., Chung E.J., Citrin D.E. (2017). Inhibition of Bcl-2/xl With ABT-263 Selectively Kills Senescent Type II Pneumocytes and Reverses Persistent Pulmonary Fibrosis Induced by Ionizing Radiation in Mice. Int. J. Radiat. Oncol..

[41] Lagares D., Santos A., Grasberger P.E., Liu F., Probst C.K., Rahimi R.A., Sakai N., Kuehl T., Ryan J.A., Bhola P. (2017). Targeted apoptosis of myofibroblasts with the BH3 mimetic ABT-263 reverses established fibrosis. Sci. Transl. Med..

[42] Fleury H., Malaquin N., Tu V., Gilbert S., Martinez A., Olivier M., Sauriol A., Communal L., Leclerc-Desaulniers K., Carmona E. (2019). Exploiting interconnected synthetic lethal interactions between PARP inhibition and cancer cell reversible senescence. Nat. Commun..

[43] Wong P., Hui A., Su J., Yue S., Haibe-Kains B., Gokgoz N., Xu W., Bruce J., Williams J., Catton C. (2015). Prognostic microRNAs modulate the RHO adhesion pathway: A potential therapeutic target in undifferentiated pleomorphic sarcomas. Oncotarget.

[44] Patra B., Lafontaine J., Bavoux M., Zerouali K., Glory A., Ahanj M., Carrier J.-F., Gervais T., Wong P. (2019). On-chip combined radiotherapy and chemotherapy testing on soft-tissue sarcoma spheroids to study cell death using flow cytometry and clonogenic assay. Sci. Rep..

[45] Bavous M., Kamio Y., Vigneux-Foley E., Lafontaine J., Najyb O., Refet E., Carrier J.-F., Gervais T., Wong P. (2021). X-Ray on chip: Quantifying therapeutic synergies between radiotherapy and anticancer drugs using soft tissue sarcoma tumor spheroids. Radiother. Oncol..

[46] Xu B., Sun Z., Liu Z., Guo H., Liu Q., Jiang H., Zou Y., Gong Y., Tischfield J.A., Shao C. (2011). Replication Stress Induces Micronuclei Comprising of Aggregated DNA Double-Strand Breaks. PLoS ONE.

[47] Ryu S.J., Oh Y.S., Park S.C. (2007). Failure of stress-induced downregulation of Bcl-2 contributes to apoptosis resistance in senescent human diploid fibroblasts. Cell Death Differ..

[48] Zhu Y., Tchkonia T., Fuhrmann-Stroissnigg H., Dai H.M., Ling Y.Y., Stout M.B., Pirtskhalava T., Giorgadze N., Johnson K.O., Giles C.B. (2016). Identification of a novel senolytic agent, navitoclax, targeting the Bcl-2 family of anti-apoptotic factors. Aging Cell.

[49] Tse C., Shoemaker A.R., Adickes J., Anderson M.G., Chen J., Jin S., Johnson E.F., Marsh K.C., Mitten M.J., Nimmer P. (2008). ABT-263: A Potent and Orally Bioavailable Bcl-2 Family Inhibitor. Cancer Res..

[50] Souers A.J., Leverson J.D., Boghaert E.R., Ackler S.L., Catron N.D., Chen J., Dayton B.D., Ding H., Enschede S.H., Fairbrother W.J. (2013). ABT-199, a potent and selective BCL-2 inhibitor, achieves antitumor activity while sparing platelets. Nat. Med..

[51] Teicher B.A., Polley E., Kunkel M., Evans D., Silvers T., Delosh R., Laudeman J., Ogle C., Reinhart R., Selby M. (2015). Sarcoma Cell Line Screen of Oncology Drugs and Investigational Agents Identifies Patterns Associated with Gene and microRNA Expression. Mol. Cancer Ther..

[52] Leverson J.D., Phillips D.C., Mitten M.J., Boghaert E.R., Diaz D., Tahir S.K., Belmont L.D., Nimmer P., Xiao Y., Ma X.M. (2015). Exploiting selective BCL-2 family inhibitors to dissect cell survival dependencies and define improved strategies for cancer therapy. Sci. Transl. Med..

[53] Bansal M., Yang J., Karan C., Menden M.P., Costello J.C., Tang H., Xiao G., Li Y., Allen J., Zhong R. (2014). A community computational challenge to predict the activity of pairs of compounds. Nat. Biotechnol..

[54] Han K., Pierce S.E., Li A., Spees K., Anderson G.R., Seoane J.A., Lo Y.-H., Dubreuil M., Olivas M., Kamber R.A. (2020). CRISPR screens in cancer spheroids identify 3D growth-specific vulnerabilities. Nat. Cell Biol..

[55] Virgone-Carlotta A., Lemasson M., Mertani H.C., Diaz J.-J., Monnier S., Dehoux T., Delanoë-Ayari H., Rivière C., Rieu J.-P. (2017). In-depth phenotypic characterization of multicellular tumor spheroids: Effects of 5-Fluorouracil. PLoS ONE.

[56] Blay J.-Y., Honoré C., Stoeckle E., Meeus P., Jafari M., Gouin F., Anract P., Ferron G., Rochwerger A., Ropars M. (2019). Surgery in reference centers improves survival of sarcoma patients: A nationwide study. Ann. Oncol..

[57] Feeney G., Sehgal R., Sheehan M., Hogan A., Regan M., Joyce M., Kerin M. (2019). Neoadjuvant radiotherapy for rectal cancer management. World J. Gastroenterol..

[58] Wang X., Yin C., Su S., Li X., Wang C., Zhang C., Liu M. (2018). Long-term effects of neoadjuvant radiotherapy, adjuvant radiotherapy, and chemotherapy-only on survival of locally advanced non-small cell lung Cancer undergoing surgery: A propensity-matched analysis. BMC Cancer.

[59] Singh P., Hoffman K., Schaverien M.V., Krause K.J., Butler C., Smith B.D., Kuerer H.M. (2019). Neoadjuvant Radiotherapy to Facilitate Immediate Breast Reconstruction: A Systematic Review and Current Clinical Trials. Ann. Surg. Oncol..

[60] Gandhi L., Camidge D.R., De Oliveira M.R., Bonomi P., Gandara D., Khaira D., Hann C.L., McKeegan E.M., Litvinovich E., Hemken P.M. (2011). Phase I Study of Navitoclax (ABT-263), a Novel Bcl-2 Family Inhibitor, in Patients With Small-Cell Lung Cancer and Other Solid Tumors. J. Clin. Oncol..

[61] Oltersdorf T., Elmore S.W., Shoemaker A.R., Armstrong R.C., Augeri D.J., Belli B.A., Bruncko M., Deckwerth T.L., Dinges J., Hajduk P.J. (2005). An inhibitor of Bcl-2 family proteins induces regression of solid tumours. Nat. Cell Biol..

[62] Wilson W.H., O’Connor O.A., Czuczman M.S., LaCasce A.S., Gerecitano J.F., Leonard J.P., Tulpule A., Dunleavy K., Xiong H., Chiu Y.-L. (2010). Navitoclax, a targeted high-affinity inhibitor of BCL-2, in lymphoid malignancies: A phase 1 dose-escalation study of safety, pharmacokinetics, pharmacodynamics, and antitumour activity. Lancet Oncol..

[63] Zhang H., Nimmer P., Tahir S.K., Chen J., Fryer R.M., Hahn K.R., Iciek L.A., Morgan S.J., Nasarre M.C., Nelson R.J. (2007). Bcl-2 family proteins are essential for platelet survival. Cell Death Differ..

[64] Vandenberg C.J., Cory S. (2013). ABT-199, a new Bcl-2–specific BH3 mimetic, has in vivo efficacy against aggressive Myc-driven mouse lymphomas without provoking thrombocytopenia. Blood.

[65] Pan R., Hogdal L.J., Benito J.M., Bucci D., Han L., Borthakur G., Cortes J., DeAngelo D.J., DeBose L., Mu H. (2014). Selective BCL-2 Inhibition by ABT-199 Causes On-Target Cell Death in Acute Myeloid Leukemia. Cancer Discov..

[66] Whittle J.R., Vaillant F., Surgenor E., Policheni A.N., Giner G., Capaldo B.D., Chen H.R., Liu H.K., Dekkers J.F., Sachs N. (2020). Dual Targeting of CDK4/6 and BCL2 Pathways Augments Tumor Response in Estrogen Receptor-Positive Breast Cancer. Clin. Cancer Res..

[67] Cang S., Iragavarapu C., Savooji J., Song Y., Liu D. (2015). ABT-199 (venetoclax) and BCL-2 inhibitors in clinical development. J. Hematol. Oncol..

[68] Roberts A.W., Davids M.S., Pagel J.M., Kahl B.S., Puvvada S.D., Gerecitano J.F., Kipps T.J., Anderson M.A., Brown J.R., A Gressick L. (2016). Targeting BCL2 with Venetoclax in Relapsed Chronic Lymphocytic Leukemia. N. Engl. J. Med..

[69] Salem A.H., Agarwal S.K., Dunbar M., Enschede S.L.H., Humerickhouse R.A., Wong S.L. (2017). Pharmacokinetics of Venetoclax, a Novel BCL-2 Inhibitor, in Patients With Relapsed or Refractory Chronic Lymphocytic Leukemia or Non-Hodgkin Lymphoma. J. Clin. Pharmacol..

[70] Lok S.W., Whittle J.R., Vaillant F., Teh C.E., Lo L.L., Policheni A.N., Bergin A.R.T., Desai J., Ftouni S., Gandolfo L.C. (2018). A Phase Ib Dose-Escalation and Expansion Study of the BCL2 Inhibitor Venetoclax Combined with Tamoxifen in ER and BCL2–Positive Metastatic Breast Cancer. Cancer Discov..

[71] Ashkenazi A., Fairbrother W.J., Leverson J.D., Souers A.J. (2017). From basic apoptosis discoveries to advanced selective BCL-2 family inhibitors. Nat. Rev. Drug Discov..

[72] Kotschy A., Szlavik Z., Murray J., Davidson J., Maragno A.L., Le Toumelin-Braizat G., Chanrion M., Kelly G.L., Gong J.-N., Moujalled D.M. (2016). The MCL1 inhibitor S63845 is tolerable and effective in diverse cancer models. Nature.

[73] Leverson J.D., Zhang H., Chen J., Tahir S.K., Phillips D.C., Xue J., Nimmer P., Jin S., Smith M.T., Xiao Y. (2015). Potent and selective small-molecule MCL-1 inhibitors demonstrate on-target cancer cell killing activity as single agents and in combination with ABT-263 (navitoclax). Cell Death Dis..

[74] Caenepeel S., Brown S.P., Belmontes B., Moody G., Keegan K.S., Chui D., Whittington D.A., Huang X., Poppe L., Cheng A.C. (2018). AMG 176, a Selective MCL1 Inhibitor, is Effective in Hematological Cancer Models Alone and in Combination with Established Therapies. Cancer Discov..

[75] Tron A.E., Belmonte M.A., Adam A., Aquila B.M., Boise L.H., Chiarparin E., Cidado J., Embrey K.J., Gangl E., Gibbons F.D. (2018). Discovery of Mcl-1-specific inhibitor AZD5991 and preclinical activity in multiple myeloma and acute myeloid leukemia. Nat. Commun..

[76] Inoue-Yamauchi A., Jeng P.S., Kim K., Chen H.-C., Han S., Ganesan Y.T., Ishizawa K., Jebiwott S., Dong Y., Pietanza M.C. (2017). Targeting the differential addiction to anti-apoptotic BCL-2 family for cancer therapy. Nat. Commun..

[77] Tahir S.K., Smith M.L., Hessler P., Rapp L.R., Idler K.B., Park C.H., Leverson J.D., Lam L.T. (2017). Potential mechanisms of resistance to venetoclax and strategies to circumvent it. BMC Cancer.

[78] Lee E.F., Harris T.J., Tran S., Evangelista M., Arulananda S., John T., Ramnac C., Hobbs C., Zhu H., Gunasingh G. (2019). BCL-XL and MCL-1 are the key BCL-2 family proteins in melanoma cell survival. Cell Death Dis..

[79] Chang B.-D., Swift M.E., Shen M., Fang J., Broude E.V., Roninson I.B. (2002). Molecular determinants of terminal growth arrest induced in tumor cells by a chemotherapeutic agent. Proc. Natl. Acad. Sci. USA.

[80] Ianzini F., Bertoldo A., Kosmacek E.A., Phillips S.L., Mackey M.A. (2006). Lack of p53 function promotes radiation-induced mitotic catastrophe in mouse embryonic fibroblast cells. Cancer Cell Int..

[81] Boden R.A., Clark M., Neuhaus S., A’hern J., Thomas J., Hayes A. (2006). Surgical management of soft tissue sarcoma in patients over 80 years. Eur. J. Surg. Oncol. (EJSO).

[82] Farshadpour F., Schaapveld M., Suurmeijer A., Wymenga A., Otter R., Hoekstra H. (2005). Soft tissue sarcoma: Why not treated?. Crit. Rev. Oncol..

[83] Lahat G., Dhuka A.R., Lahat S., Lazar A.J., Lewis V.O., Lin P.P., Feig B., Cormier J.N., Hunt K.K., Pisters P.W.T. (2009). Complete Soft Tissue Sarcoma Resection is a Viable Treatment Option for Select Elderly Patients. Ann. Surg. Oncol..

[84] Blumenfeld P., Sen N., Abrams R.A., Wang D. (2016). Advances in Radiation Therapy for Primary and Metastatic Adult Soft Tissue Sarcomas. Curr. Oncol. Rep..

[85] Chen Z., Wu Z., Ning W. (2019). Advances in Molecular Mechanisms and Treatment of Radiation-Induced Pulmonary Fibrosis. Transl. Oncol..

[86] Schafer M.J., White T.A., Iijima K., Haak A.J., Ligresti G., Atkinson E.J., Oberg A.L., Birch J., Salmonowicz H., Zhu Y. (2017). Cellular senescence mediates fibrotic pulmonary disease. Nat. Commun..

[87] Campeau E., Ruhl V.E., Rodier F., Smith C.L., Rahmberg B.L., Fuss J.O., Campisi J., Yaswen P., Cooper P.K., Kaufman P.D. (2009). A Versatile Viral System for Expression and Depletion of Proteins in Mammalian Cells. PLoS ONE.

[88] Lansu J., Bovée J.V., Braam P., Van Boven H., Flucke U., Bonenkamp J.J., Miah A.B., Zaidi S.H., Thway K., Bruland Ø.S. (2020). Dose Reduction of Preoperative Radiotherapy in Myxoid Liposarcoma: A Nonrandomized Controlled Trial. JAMA Oncol..

