# Supplementary material for: Senolytic Targeting of Bcl-2 Anti-Apoptotic Family Increases Cell Death in Irradiated Sarcoma Cells
Source: Cancers (Basel). 2021 Jan 21;13(3):386. doi: 10.3390/cancers13030386 (PMC7866159; doi:10.3390/cancers13030386)
Supplement: Supplementary file 1 [file cancers-13-00386-s001.pdf]

cancers

MDPI

Supplementary Materials: Senolytic Targeting of Bcl-2 Anti-Apoptotic Family Increases Cell Death in Irradiated Sarcoma Cells

Julie Lafontaine, Guillaume B. Cardin, Nicolas Malaquin, Jean-Sébastien Boisvert, Francis Rodier and Philip Wong

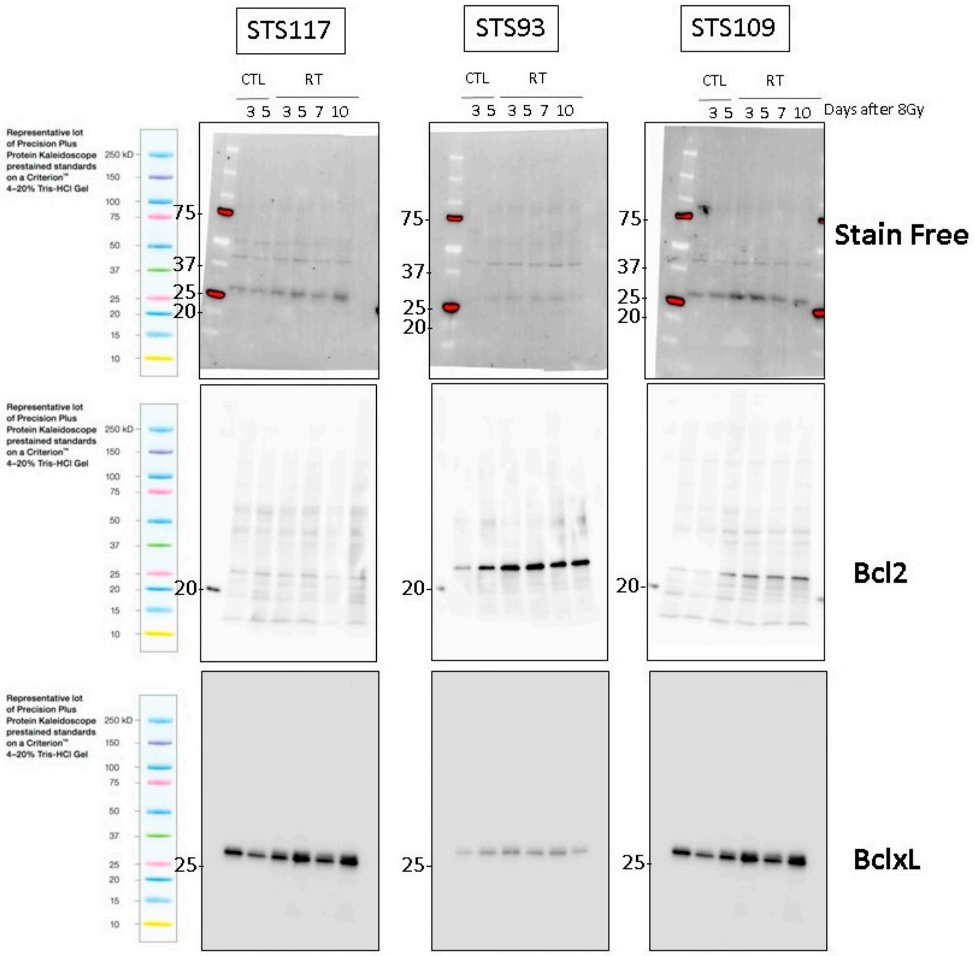

Citation: Lafontaine, J.; Cardin, G.B.; Malaquin, N.; Boisvert, J.-S.; Rodier, F.; Wong, P. Senolytic Targeting of Bcl-2 Anti-Apoptotic Family Increases Cell Death in Irradiated Sarcoma Cells. *Cancers* **2021**, *13*, 386. https://doi.org/10.3390/cancers13030386

Received: 10 December 2020
Accepted: 18 January 2021
Published: 21 January 2021

Publisher's Note: MDPI stays neutral with regard to jurisdictional claims in published maps and institutional affiliations.

Copyright: © 2021 by the authors. Submitted for possible open access publication under the terms and conditions of the Creative Commons Attribution (CC BY) license (http://creativecommons.org/licenses/by/4.0/).

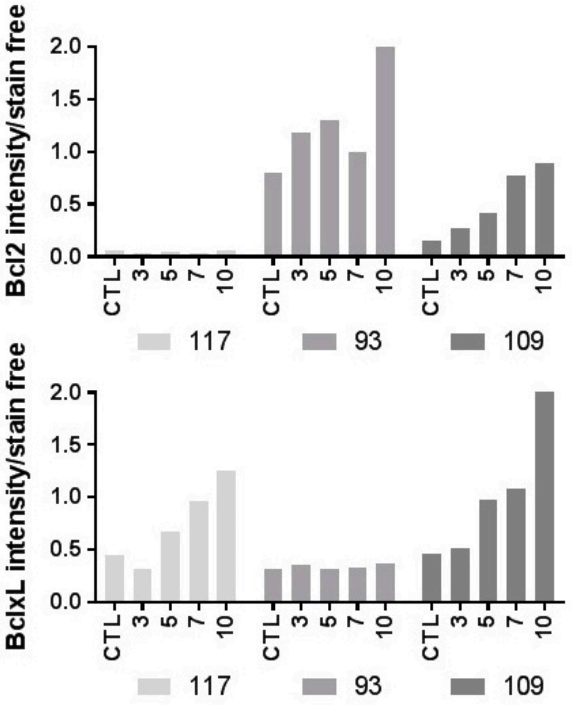

Figure 1. Western blot and densitometry analysis of BCL2 and BCL-XL. STS cells were treated with RT (8Gy) and protein level was measured over days. Original image of western blots and stain free membranes. Intensity ratio represent intensity of protein band normalized to the corresponding stain free value. In graph, CTL is the mean of the ratio from say 3 and 5.
